# Supplementary material for: Decreased Fatty Acid Transporter FABP1 and Increased Isoprostanes and Neuroprostanes in the Human Term Placenta: Implications for Inflammation and Birth Weight in Maternal Pre-Gestational Obesity
Source: Nutrients. 2021 Aug 12;13(8):2768. doi: 10.3390/nu13082768 (PMC8398812; doi:10.3390/nu13082768)
Supplement: Supplementary file 1 [file nutrients-13-02768-s001.zip › nutrients-1329500-supplementary.pdf]

Supplementary File S1. Supplementary methods

1. Assessment of Dietary Intake

Two 24-h recall (24hR) were collected in the 3rd trimester of pregnancy: one face-to-face and another up to 2 days later, by a phone interview. This approach was already validated by Barbieri et al. (2016) to access the dietary intake of Brazilian pregnant women.

Trained nutritionists systematized the portions and home measures, as well as the insertion of dietary data into the database. Nutrient intake was analyzed by the software DietBox® v.4.0, Rio de Janeiro, Bazil, using the Tabela Brasileira de Composição de Alimentos [1] and Instituto Brasileiro de Geografia e Estatística [2] database. Fatty acid intake was analyzed using the United States Department of Agriculture database.

Habitual nutrient intake was estimated by the Multiple Source Method, a statistical method that considers intra- and inter-individual variation over the same period using at least two different dietary inputs (e.g., dietary recalls and/or food frequency questionnaire [3]. It has been shown to be adequate to estimate usual intake during pregnancy [4].

2. Placental Sampling

Placentas were sampled from their maternal surface after removal of 1–2 mm of the basal plate. Villous samples 1–2 cm^3 were cut from 4 random sites and washed twice in phosphate-buffered saline at 4 °C. Smaller fragments for protein (~ 50 mg); transcripts (~ 10 mg) and lipid (50–100 mg) analyses were placed into cryovials and immediately frozen in liquid nitrogen. All samples were stored at −80 °C until analysis.

3. Fatty Acid Transporter Proteins in Placental Tissue

3.1. Quantitative Real-Time PCR

Total RNA from placental tissue (10 mg) was extracted using TRIzol® and PureLink® RNA Mini Kit and addition of PureLink® DNAse (Invitrogen, Thermo Fisher Scientific, Waltham, MA, USA), according to the manufacturer's instructions. The concentration of RNA was determined using a NanoDrop™ spectrophotometer (Thermo Fisher Scientific, Waltham, MA, USA). cDNA synthesis was accomplished using High-Capacity cDNA Reverse Transcription® (Applied Biosystems, Thermo Fisher Scientific, Waltham, MA, USA). Quantitative real-time PCR (qPCR) was performed using TaqMan ™ Universal PCR Master Mix (Applied Biosystems, Thermo Fisher Scientific, Waltham, MA, USA) with the following primers from TaqMan™ Thermo Fisher Scientific, MA, USA Hs00195812 (EL), Hs00155026 (FABP1) and Hs00997360 (FABP3). Glyceraldehyde-3-phosphate dehydrogenase (GAPDH) was used as the housekeeping gene and transcripts were calculated using the threshold cycle 2-ΔΔCT method [5].

3.2. Western Blotting

Placental fragments (50 mg) were prepared in lysis buffer containing 100 mM Tris (pH 7.5), 10 mM EDTA, 10% SDS, 10%, 100 mM NaF, 10 µM sodium pyrophosphate, 10 µM Na_3VO_4. Tissues were homogenized in a IKA®RW20 instrument (IKA Brasil, Sao Paulo,

Brazil). After homogenization was complete, 10 % TritonX-100 was added to the samples. Placental lysates were prepared in gel loading buffer containing Laemmli buffer (Biorad Laboratories Inc, Hercules, CA, USA) (70 mM Tris-HCl (pH 6.8), 50 mM DTT, 1% SDS, 10% glycerol and 0.005% bromophenol blue) and heated at 70 °C for 5 min. Samples were loaded and resolved in SDS-PAGE gels in a mini-PROTEAN® System (BioRad Laboratories Inc, Hercules, CA, USA) and blotted onto nitrocellulose. The nitrocellulose membrane was blocked with 1% bovine serum albumin in Tris-HCl 10 mM, 150 mM NaCl, Tween-20 50 μL/mL and incubated overnight with the primary antibody at 4 °C and 2 h at room temperature. The membrane was then incubated with HRP-conjugated secondary antibody (HRP; ABCAM, Cambridge, United Kingdom) for 1 h at room temperature.

Protein bands were analysed by enhanced chemiluminescence (Biorad Laboratories Inc, Hercules, CA, USA) and the films were scanned in a flat-bed scanner (ImagiQuant LAS 4000, Sinapse Biotecnologia, Sao Paulo, Brazil) and band intensities were determined from two different exposures (within the linear detection range) with background subtraction using Image Studio Lite v5.2 software (Li-cor biosciences, Biosciences do Brasil, Sao Paulo, Brazil).

Primary antibodies for EL (MBS2013720) from My BioSource, Inc. (San Diego, CA, USA), FABP1 (AB7366) and FABP3 (AB16916) from ABCAM (Cambridge, United Kingdom) were used in the following dilutions, respectively: 1:1000 EL, 1:500 and 1:500. Loading control was performed with anti-β-actin antibody (Sigma-Aldich, Saint Louis, MO, USA, SAB5500001).

4. Maternal Lipoprotein Profile

Lipoproteins were assayed using commercial kits (Triglycerides and Monoreagent Cholesterol, Direct LDL and BIOCLIN® Enzymatic HDL Cholesterol, Sigma-Aldich, Saint Louis, MO, USA), and absorbance measured at 500, 540 and 500 nm, respectively (SpectraMax® Plus 384 spectrophotometer, Biosciences do Brasil, Sao Paulo, Brazil). The concentration of very low density lipoprotein (VLDL-c) was estimated using the formula suggested by Friedewald, Levy and Fredrickson [6].

5. Placental Lipid Profile Analysis

Lipids were extracted using a modified Folch method [7]. Briefly, 0.35 mL of cold methanol and 0.35 mL of chloroform were added to vials containing ~ 50 mg of frozen placental tissue and one metallic bead and were disrupted in a single batch (Tissue Lyser II, Qiagen; 2 × 2.5 min, 17 s^{-1}, Hilden, Germany). Next, following addition of 0.35 mL of chloroform and 0.15 mL of ultrapure water (Chromasolv; Honeywell, Reidel-de Haën, Thermo Fisher Scientific, Waltham, MA, USA), samples were thoroughly vortexed for 30 seconds and centrifuged (17,000 × *g*, 10 min). The lower phase containing the lipid extract was transferred to glass vials, dried under a gentle stream of nitrogen and stored at −80 °C until further analysis.

The dried chloroform phase was suspended in 50 μL methanol containing the internal standards (25 deuterated lipids, representatives of phosphatidic acid, phosphatidylcholines, phosphatidylethanolamines, glycerophospholipids, phosphatidylinositols, phosphatidylserine, sphingomyelin, ceramides, triacylglycerols and fatty acids (Supplementary Table s1) and 75 μL of

a solution of isopropanol:acetonitrile:water (IPA:ACN:H_2O, 2:1:1, v/v). Quality control (QC) samples consisted of a pooled sample containing 10 μL aliquots of each sample. All samples were analysed in positive and negative mode. For positive mode, the samples and QC were diluted 1:10 with the IPA:ACN:H_2O (2:1:1) solution.

5.1. Ion Mobility QTOF LC/MS Lipid Profile Analysis of Placenta Samples

An Agilent 6560 Ion mobility Quadrupole Time-of-Flight (DTIM-QTOF) LC-MS system coupled with an Agilent 1290 UHPLC system was used to combine separation power and selectivity of LC, DTIM, and MS techniques. The Dual Agilent Jet Stream electrospray ionization source was operated separately in positive and negative ion mode.

The lipid extract was separated in a ACQUITY CSH C18 column (100 × 2.1 mm and 1.7 μm particle diameter, Waters) conditioned at 55 °C. The mobile phase consisted of: (A) 10 mM ammonium formate solution in 40 % ultrapure water and 60% acetonitrile and (B) 10 mM ammonium formate solution in 90% isopropanol, and 10 % acetonitrile (v/v). The mobile phase was pumped at a flow rate of 400 μL/min starting at 60% of A and 40% of B. Solvent B was increased linearly to 43% in 2 min, to 50% at 2.1 min, to 54% at 12 min and then to 99% in 18 min. At 18.1 min solvent B was brought back to the initial conditions and remained at this percentage for 1.9 min. The column was re-equilibrated for 3 min at 40% solvent B.

An Agilent tuning solution was injected before the analysis to tune the instrument in the m/z range 100–1700, and before every 10 samples to perform CCS re-calibration. During samples acquisition, an Agilent reference mix was constantly injected for mass re-calibration. The Agilent Mass Hunter LC-MS acquisition console was used for data acquisition.

5.2 Positive Ion Mode

The electrospray capillary potential was set to 60 V, the needle at 20 kV. Nitrogen gas at 5 L/min (set at 48 mTorr) and 375 °C was used as drying gas for solvent evaporation; sheath gas was set at 275 °C and 12 L/min flow rate. Full-scan spectra were obtained in the ranges of 50–1200 amu, scan time of 0.20 sec., scan width of 0.70 amu, and detector set at 2950 V.

5.3. Negative Ion Mode

The electrospray capillary potential was set to 60 V, and the needle at 20 kV. Nitrogen gas at 5 L/min (set at 48 mTorr) and at 375 °C was used as drying gas for solvent evaporation, sheath gas was set at 275 °C and 12 L/min flow rate. Full-scan spectra were obtained in the ranges of 50–1200 amu, 0.20 sec can time, 0.70 amu scan width, and detector set at 2950 V.

5.4. Chromatogram Pre-Processing

Data pre-processing, including mass and CCS re-calibration and feature finding, was carried out using the packages IM-MS Reprocessor, IM-MS Browser and Mass Profiler from the MassHunter Suite (vB.08.00, Agilent Technologies, Santa Clara, CA, USA).

The resulting data matrices were processed using a KNIME (https://www.knime.com) pipeline comprising both KNIME native nodes and integrated R scripts. QC-based feature filtering with missing value 50 and relative standard deviation threshold 0.2 was performed

to eliminate noise/background signals and to remove features with poor repeatability. Imputation of missing values was performed using a Key-nearest neighbour (KNN) approach based on the R library impute. Feature annotation was performed based on the AccurateMassSearch node of the OpenMS library [8].

6. Isoprostanoids in Placental Tissue

Isoprostanoids in placenta were determined based on a microLC–MS/MS method [9]. Briefly, lipid extracts were obtained by the Folch method [7], which were then mixed with a mixture of internal standards, followed by alkaline hydrolysis. The metabolites were concentrated via solid phase extraction in weak-anion exchange cartridges, and then analyzed by microLC–MS/MS. Mass spectrometry analysis was performed in a AB Sciex QTRAP 5500 (AB Sciex, MA, USA) with an electrospray ionization source operated in negative mode. Detection of the fragmentation ion products from each deprotonated molecule was performed in the multiple reaction monitoring mode. Quantification of isoprostanoids was performed with the MultiQuant v3.0 software (AB Sciex, Framingham, MA, USA), by the ratio between each specific metabolite peak area and the internal standard peak area calibrated by curves of the metabolite standards peak area versus the internal standard peak area.

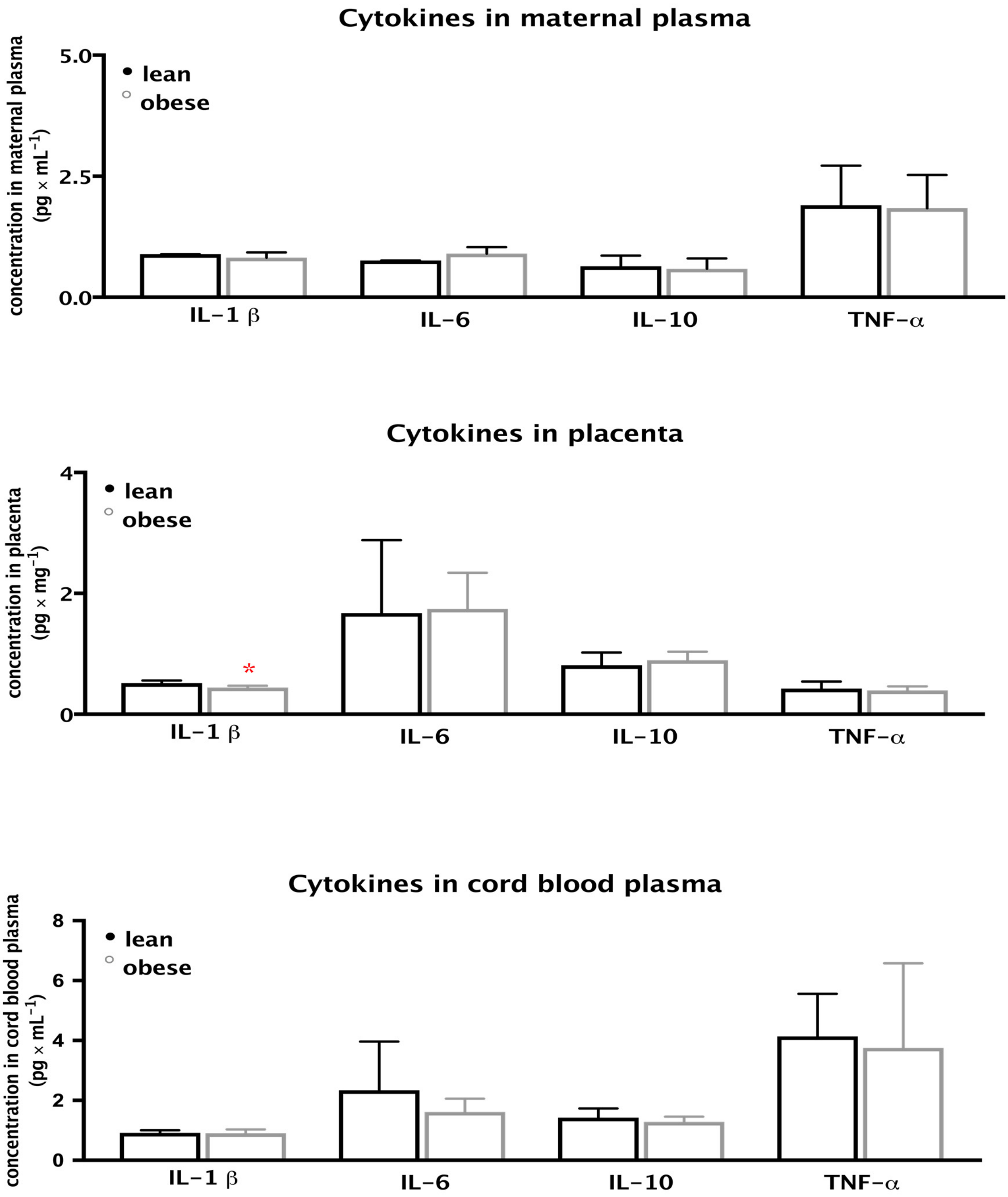

Supplementary Figure S1. Cytokines in maternal plasma, in the placenta and in cord blood plasma from lean (black bars) and obese (gray bars) women. * Significantly different from the lean group; $p < 0.05$ (Mann–Whitney test). IL-1β: Interleukin-1β; IL-6: Interleukin-6; IL-10: Interleukin-10; TNF-α: Tumour Necrosis Factor-α.

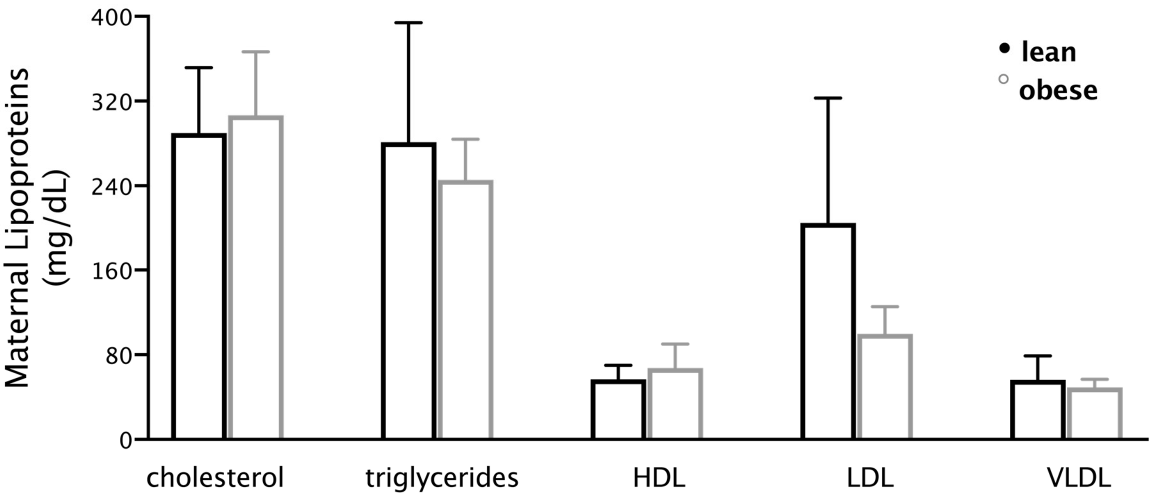

Supplementary Figure S2. Total cholesterol, lipoproteins and triglycerides in plasma of lean (black bars) and obese (gray bars) women.

Supplementary Table S1. Internal standards used in the Ion Mobility QTOF LC/MS lipid profile analysis.

Name	Trivial Name	Class	Store
N-palmitoyl-d31-D-erythro-sphingosine (C16-d31 Ceramide)	C16-d31 Ceramide	Cer	avanti
1-palmitoyl-d31-2-oleoyl-sn-glycero-3-phosphate (sodium salt) (16:0-d31-18:1 PA)	16:0-d31-18:1 PA	PA	avanti
1-palmitoyl(D31)-2-oleyl-sn-glycero-3-phosphocholine (16:0-d31-18:1 PC)	16:0-d31-18:1 PC	PC	avanti
1-palmitoyl(D31)-2-oleyl-sn-glycero-3-phosphoethanolamine (16:0-d31-18:1 PE)	16:0-d31-18:1 PE	PE	avanti
1-palmitoyl-d31-2-oleoyl-sn-glycero-3-[phospho-rac-(1-glycerol)] (sodium salt) (16:0-d31-18:1 PG)	16:0-d31-18:1 PG	PG	avanti
1-palmitoyl-d31-2-oleoyl-sn-glycero-3-phosphoinositol (ammonium salt) (16:0-d31-18:1 PI)	16:0-d31-18:1 PI	PI	avanti
1,2-dimyristoyl-d54-sn-glycero-3[phospho-L-serine] (sodium salt)(14:0 PS-d54)	14:0 PS-d54	PS	avanti
N-palmitoyl(D31)-d-erythro-sphingosylphosphorylcholine (16:0-d31 SM)	16:0-d31 SM	SM	avanti
1,2-dipalmitoyl-d62-sn-glycero-3-[phospho-L-serine] (sodium salt)	16:0 PS-d62	PS	avanti
cholesteryl-2,2,3,4,4,6-d6-octadecanoate (18:0-d6 CE)	18:0-d6 CE	Cer	qmx
Pentadecanoic-d29 Acid (15:0-d29 FA)	15:0-d29 FA	FA	qmx
Heptadecanoic-d33-acid (17:0-d33 FA)	17:0-d33 FA	FA	qmx
Eicosanoic-d39 Acid (20:0-d39 FA)	20:0-d39 FA	FA	qmx
Tetradecylphosphocholine-d42 (14:0-d29 LPC-d13)	14:0-d29 LPC-d13	LysoPC	qmx
Glyceryl Tri(pentadecanoate-d29) (45:0-d29 TG)	45:0-d29 TG	TAG	qmx
glyceryl-tri(hexadecanoate-d31) (48:0-d31 TG)	48:0-d31 TG	TAG	qmx
Glyceryl Tri(octadecanoate-d35) (54:0-d35 TG)	54:0-d35 TG	TAG	qmx
arachidonic acid d8	arachidonic acid d8	PUFA	Cayman Chemicals
linoleic acid d4	linoleic acid d4	PUFA	Cayman Chemicals
11(12)-EET-d11	11(12)-EET-d11	oxFA[a]	Cayman Chemicals
Prostaglandin E2-d4	Prostaglandin E2-d4	oxFA[a]	Cayman Chemicals
12-HETE-d8	12-HETE-d8	oxFA[a]	Cayman Chemicals
TxB2-d4	TxB2-d4	oxFA[a]	Cayman Chemicals
LTB2-d4	LTB2-d4	oxFA[a]	Cayman Chemicals

[a]oxFA = oxidised fatty acid.

Supplementary Table S2. Energy and Nutrient intake of women participating in the study.

Energy and Nutrients	Intake [a]		Inadequacy [b]		Reference Value (Daily Intake)
	Lean ($n = 6$)	Obese ($n = 6$)	Lean	Obese	
Energy (kcal)	1882 (1324–2040)	1707 (1462–2263)	-	-	-
Carbohydrates (%energy)	56.27 (53.98–60.63)	54.26 (52.95–57.02)	0%	0%	45–65%
Protein (%energy)	15.99 (13.98–18.14)	17.63 (16.07–21.10)	0%	0%	10–35%
Lipids (%energy)	27.88 (26.71–29.28)	26.69 (25.77–27.89)	0%	0%	20–35%

24hR-MSM: four 24-hour recalls were used, and data was assessed by the *Multiple Source Method.* [a] Nutrients are expressed as median and interquartile range (p25-75) and compared by Mann-Whitney test. [b] Frequency of women with inadequate intake, according to the reference value, compared by Fisher`s exact test. [c] Acceptable macronutrient distribution ranges according to % energy intake [10]. No significant difference between groups; $p > 0.05$. - not applicable.

References

1. NEPA/UNICAMP. Tabela brasileira de composição de alimentos. Campinas., 2011.
2. IBGE. Pesquisa de Orçamentos Familiares 2008/2009: Tabelas de Composição Nutricional dos Alimentos Consumidos no Brasil. Ministério do Planejamento, Orçamento e Gestão, Rio de Janeiro-RJ, Brazil, 2011, ISBN 978-85-240-4194-5.
3. Harttig, U.; Haubrock, J.; Knüppel, S.; Boeing, H.; EFCOVAL Consortium. The MSM program: web-based statistics package for estimating usual dietary intake using the Multiple Source Method. *Eur. J. Clin. Nutr.* 2011, *65*, doi: 10.1038/ejcn.2011.92.
4. Sartorelli, D.; Barbieri, P.; Perdoná, G. Fried food intake estimated by the multiple source method is associated with gestational weight gain. *Nutr. Res.* 2014, *34*, 667–673.
5. Livak, K. J.; Schmittgen, T.D. Analysis of relative gene expression data using real-time quantitative PCR and the 2(-Delta Delta C(T)) Method. *Methods* **2001**, *25*, 402–408, doi: 10.1006/meth.2001.1262.
6. Friedewald, W.T.; Levy, R.I.; Fredrickson, D.S. Estimation of the concentration of low-density lipoprotein cholesterol in plasma, without use of the preparative ultracentrifuge. *Clin. Chem.* **1972**, *18*, 499–502, doi: 10.1093/clinchem/18.6.499.
7. Folch, J.; Lees, M.; Sloane, S.G.H. A simple method for the isolation and purification of total lipides from animal tissues. *J. Biol. Chem.* **1957**, *226*, 497–509, doi: 10.1016/S0021-9258(18)64849-5.
8. Liggi, S.; Hinz, C.; Hall, Z.; Santoru, M.L.; Poddighe, S.; Fjeldsted, J.; Atzori, L.; Griffin, J.L. KniMet: a pipeline for the processing of chromatography-mass spectrometry metabolomics data. *Metabolomics* **2018**, *14*, 52, doi: 10.1007/s11306-018-1349-5.
9. Dupuy, A.; Le Faouder, P.; Vigor, C.; Oger, C.; Galano, J.M.; Dray, C.; Lee, J.C.; Valet, P.; Gladine, C.; Durand, T.; Bertrand-Michel, J. Simultaneous quantitative profiling of 20 isoprostanoids from omega-3 and omega-6 polyunsaturated fatty acids by LC-MS/MS in various biological samples. *Anal Chim. Acta* **2016**, *921*, 46–58, doi: 10.1016/j.aca.2016.03.024.
10. Institute of Medicine. Dietary Reference Intakes for Energy, Carbohydrate, Fiber, Fat, Fatty Acids, Cholesterol, Protein, and Amino Acids. The National Academies Press: Washington, DC, USA, 2005; https://doi.org/10.17226/10490.
